# Effects of epinephrine on heart rate variability and cytokines in a rat sepsis model

**DOI:** 10.17305/bjbms.2018.3565

**Published:** 2020-02

**Authors:** Yun-Te Chang, Wei-Chun Huang, Chin-Chang Cheng, Meng-Wei Ke, Jung-Shun Tsai, Yao-Min Hung, Neng-Chyan Huang, Mu-Shun Huang, Shue-Ren Wann

**Affiliations:** 1 Department of Emergency Medicine, Kaohsiung Veterans General Hospital, Kaohsiung City, Taiwan, Republic of China; 2 Department of Nursing, Yuh-Ing Junior College of Health Care & Management, Kaohsiung City, Taiwan, Republic of China; 3 Department of Physical Therapy, Shu-Zen Junior College of Medicine and Management, Kaohsiung City, Taiwan, Republic of China; 4 School of Medicine, National Yang-Ming University, Taipei City, Taiwan, Republic of China; 5 Cardiovascular Medical Center, Kaohsiung Veterans General Hospital, Kaohsiung City, Taiwan, Republic of China; 6 Department of Medical Education and Research, Kaohsiung Veterans General Hospital, Kaohsiung City, Taiwan, Republic of China

**Keywords:** Sepsis, heart rate variability, HRV, epinephrine, EPI, inflammation, β-blocker, rats

## Abstract

Catecholamines have both anti-inflammatory and vasoactive properties. A decreased cardiac response to catecholamines has been associated with a high risk of death in sepsis and septic shock. The aim of this study was to investigate the effects of epinephrine (EPI) on heart rate variability (HRV) and autonomic balance, as well as cytokine levels, in a rat sepsis model. Thirty-six male Sprague-Dawley rats were assigned to 4 experimental groups and 2 control groups of 6 rats each. The rats in the experimental groups were inoculated with a lipopolysaccharide (LPS, endotoxin) to establish a sepsis model. Group A received only LPS; group B received LPS, antecedent EPI and the nonselective β-blocker propranolol; group C received LPS and antecedent EPI; and group D received LPS, antecedent EPI and the selective β1-blocker esmolol. One control group received EPI and the other received saline placebo. Heart rate variability (HRV) was analyzed and tumor necrosis factor-α (TNF-α), interleukin-6 (IL-6) and interleukin-1β (IL-1β) levels were measured. Measurements were carried out at baseline, at 0 hour after EPI infusion, and at 0.5, 2, and 4 hours after LPS inoculation. There were significant differences in HRV and cytokine levels between the groups, indicating that LPS infusion caused autonomic imbalance. Antecedent EPI significantly decreased the level of TNF-α in group C compared with group A in which TNF-α level peaked at 2 hours and then declined. Propranolol (group B) but not esmolol (group D) administration resulted in elevated TNF-α levels, comparable to those observed in group A. In conclusion, antecedent administration of EPI in a rat sepsis model inhibits the production of TNF-α possibly via the β_2_-adrenoceptor.

## INTRODUCTION

Despite advances in the treatment of sepsis, including its most severe form septic shock, the mortality rate ranges from 30% to as high as 76% [1,2]. In critical care patients, sepsis syndrome and multiple organ dysfunction syndrome (MODS) are the leading cause of death [3]. Bedside experience and clinical studies showed that dopamine sensitivity was associated with lower mortality rates in septic shock patients, suggesting that blunted cardiovascular responsiveness to catecholamines is a predictive factor for mortality in sepsis and septic shock [4]. In addition, sepsis is characterized by systemic inflammation which has been attributed to altered regulation of anti-inflammatory mechanisms and adverse outcomes [1,5].

Clinical assessment of inflammation (e.g., based on vital signs and biochemical analysis) lack sufficient sensitivity and specificity to guide therapeutic intervention, and might be further improved by dynamic quantification of the functional capacity of host organ systems. Heart rate variability (HRV) analysis, including low-frequency (LF) spectral power of HR as an index of sympathetic nervous activity, high-frequency (HF) as an index of parasympathetic nervous activity and the LF-to-HF ratio, can serve as approximate measures of autonomic function. These measures are obtained by non-invasive methods and might reflect real-time changes in physiological status [6,7].

Catecholamines have major effects on the cardiovascular and immune system. The physiological actions of catecholamines are mediated by α- and β-adrenoceptors (AR) [8]. The two major types of α-ARs are α1 and α2, each of which is further divided into at least three subtypes (α1A, 1B and 1D and α2A, 2B and 2C) [8,9]. Similarly, β-ARs are divided into β1, β2, and β3 subtypes, and all three are involved in β-AR-induced vasodilation [10]. The receptor profile of the catecholamine epinephrine (EPI, also known as adrenaline) is complex, and its pharmacological effects are mediated by peripheral β1-, β2-, and α1-ARs. Catecholamines have both anti-inflammatory and vasoactive properties [11]. Acute EPI administration was suggested to be protective against early inflammatory changes during the systemic inflammation induced by endotoxin, but also to diminish vagal nerve responsiveness to subsequent stimuli, as demonstrated in healthy volunteers administered with either saline + lipopolysaccharide (LPS, endotoxin) or LPS + antecedent EPI [12]. Catecholamines have been reported to inhibit the secretion of pro-inflammatory cytokines such as tumor necrosis factor alpha (TNF-α) [13], interleukin 1 beta (IL-1β) and IL-8 [12], as well as to increase the production of anti-inflammatory cytokine IL-10 [12,14]. The stimulation of macrophages by LPS *in vitro* leads to activation of β-ARs which downregulate pro-inflammatory cytokines, particularly TNF-α, IL-1β and IL-6, and upregulate anti-inflammatory IL-10 [8]. The main β-AR expressed by macrophages is β2; however, β1 may also be involved in the modulation of macrophage function [8].

In our previous study [2], we demonstrated that the concentrations of the inflammatory mediators IL-6 and TNF-α were the highest 2 hours after *Escherichia coli* (*E. coli*) inoculation in irradiated immunocompromised rats and subsequently were decreased at 6 hours; on the contrary, IL-1β levels stayed approximately the same at all time points examined. Both major HRV components, LF and HF, were decreased during the early hyperdynamic phase after bacteria inoculation. Similarly, HR parameters decreased two hours after *E. coli* inoculation, indicating that impaired autonomic nervous control of the cardiovascular system is due to sepsis [2].

In the current study, we hypothesized that EPI infusion will affect the cardiovascular and immune response in sepsis. Specifically, our aim was to assess whether antecedent EPI modulates the HRV and autonomic balance, as well as cytokine levels, in LPS-induced sepsis in rats. In addition, we investigated the role of β1- and β2-ARs in mediating EPI effects during sepsis using selective and nonselective β-blocking agents.

## MATERIALS AND METHODS

### Animals

The study included 36 male Sprague-Dawley rats weighing 450–500 g. The approval for this study was obtained from the Institutional Animal Care and Use Committee (IACUC) of Kaohsiung Veterans General Hospital, Taiwan, Republic of China.

### Animal preparation

The animals were anesthetized by intramuscular injection of zoletil 50 (1:1 ratio of tiletamine hydrochloride and zolazepam hydrochloride), in a dose of 20–40 mg/kg or 0.4–0.8 mL/kg. The trachea was orally intubated with a 14-gauge cannula mounted on a blunt needle with a 145° angled tip (Abbocath-T, Abbott Hospital, North Chicago, IL, USA), as previously described [15]. The animals breathed spontaneously in room air. Electrocardiogram (ECG) lead II was continuously monitored and recorded. Core blood temperature was measured with a thermocouple microprobe (#9030-12-34, Columbus Instruments, Columbus, OH, USA), which was advanced through the right femoral artery into the thoracic aorta. A 23-gauge polyethylene catheter (PE 50) was advanced through the left femoral vein for EPI infusion and another PE 50 was advanced through the right common carotid artery for blood sampling.

### Inoculation material

LPS, which is derived from the cell wall of most Gram-negative bacteria, was purchased from Sigma (Sigma-Aldrich, St. Louis, MO, USA).

### Experimental protocol and measurements

**FIGURE 1:**
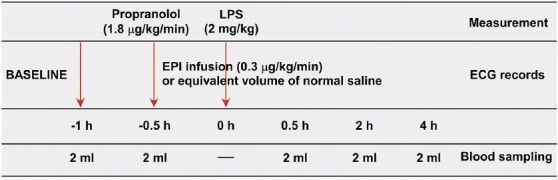
Experimental protocol used in the study. Propranolol or esmolol were given 1 hour before lipopolysaccharide (LPS) inoculation, and epinephrine (EPI) infusion was begun 0.5 hour before LPS inoculation. Measurements were obtained until 4 hours after LPS inoculation.

The experimental protocol is shown in Figure 1. After being anesthetized, the rats were randomized into four experimental groups and two control groups of six animals each. The rats in the experimental groups were inoculated with LPS to establish a sepsis model. EPI (0.3 µg/kg/minute) or saline infusion was administered intravenously during surgery 30 minutes before the LPS inoculation, and was continued for additional 4 hours. Group A received LPS (2 mg/kg, endotoxin) without EPI; Group B received LPS, antecedent EPI (0.3 µg/kg/minute) and nonselective (β1- and β2-) blocker propranolol (1.8 µg/kg/minute) infusion; Group C received LPS and antecedent EPI; and Group D received LPS, antecedent EPI and selective β1-blocker esmolol (3 µg/kg/minute) infusion. One control group received EPI (without LPS) and the other received saline placebo. Vital signs, including HR and mean arterial blood pressure (MBP), were recorded for the entire 6-hour period from the PC-based data during the experiment. Electrocardiography was recorded continuously during experiment and blood cytokine levels (TNF-α, IL-1β, and IL-6) were analyzed at baseline (0.5 hours before EPI infusion), at 0 hour after EPI infusion, and at 0.5, 2, and 4 hours after LPS inoculation. Blood lactate level at 0 and 2 hours after LPS inoculation was also evaluated.

### Assessment of HRV

Baseline of HRV parameters were measured and recorded after surgery and during the experiment. Each recording interval of HRV parameters consisted of two consecutive 5-minute periods. Continuous electrocardiography was used to determine the HRV parameters and interbeat intervals at a rate of 256 samples per second. In addition, both time- and frequency-domain measures of HRV were analyzed. Time-domain measures included: 1) measuring the total HRV and overall system adaptability - standard deviation of the average NN intervals (beat-to-beat) for each 5-minute period (SDANN); 2) root mean square of successive RR interval difference (RMSSD) which is primarily influenced by vagus nerve; and 3) percentage of successive RR intervals that differ by greater than 50 ms (pNN50), usually associated with respiratory sinus arrhythmia. On the other hand, frequency domain measures included: 1) very low frequency variability (VLF; 0.005–0.05 Hz) related to the thermoregulation and sympathetic contribution to vascular regulation; 2) low-frequency variability (LF; 0.05–0.15 Hz), correlated with both parasympathetic and sympathetic activation; and 3) high-frequency variability (HF; 0.15–0.4 Hz) associated with parasympathetic and vagal tone; as well as 4) the ratio of LF/HF. Herein, the LF/HF ratio was considered to be related to the balance between the sympathetic and parasympathetic nerves [16-18].

The record of instantaneous heart rate was produced from a continuous electrocardiographic record. Each QRS complex and the interval between adjacent QRS complexes caused by sinus node depolarization (normal-to-normal intervals) were detected and tabulated [17]. Before interbeat intervals were calculated using Chart Modules software (AD Instruments Pty Ltd., Bella Vista, NSW, Australia), visual inspection and interpolation was used to reduce noise artifact and irregular heartbeats. Each epoch was analyzed as previously reported [19] and events such as skipped beats, extra-systolic heart beats, and arrhythmias were excluded. In addition, the Fast Fourier transformation algorithm was used to calculate power spectral density [17,20]. All collected signals (standard ASCII format) were exported to Excel for analysis and graphics.

### Blood sample analysis

Blood samples (0.5 mL per rat) were collected at the following time points: baseline (0.5 hour before EPI infusion), 0 hour (after EPI infusion), 0.5, 2, and 4 hours after LPS inoculation. Plasma levels of inflammatory markers TNF-α, IL-6, and IL-1β were evaluated by enzyme-linked immunosorbent assay (ELISA) [21]. Lactate levels at 0 and 2 hours after LPS inoculation were also measured.

At the end of experiment, animals were euthanized with an intraperitoneal injection of pentobarbital sodium (150 mg/kg). All animals were examined at autopsy for evidence of gross injury to the chest and abdominal organs due to the experimental procedures.

### Statistical analysis

Data were presented as the mean ± standard deviation (SD). One-way analysis of variance (ANOVA) with Scheffe’s multi-comparison test was used to compare differences in measurements between groups at each time point. The differences in measurements between two time points within each group were analyzed by the paired *t*-test. The level of statistical significance was set at two-sided *p* < 0.05. Statistical analyses were performed using IBM SPSS Statistics for Windows, Version 22.0. (IBM Corp., Armonk, NY, USA).

## RESULTS

### HRV analysis

#### HR

**FIGURE 2:**
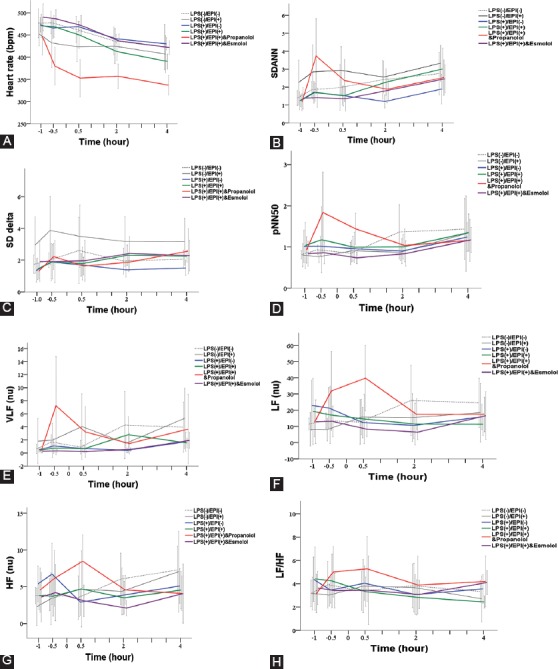
Changes in HRV parameters in LPS-induced rat sepsis model, and in relation to EPI and propranolol/esmolol administration. A total of 36 male Sprague-Dawley rats were randomized into two control groups and four experimental groups of six animals each. Black dotted line denotes saline placebo group: LPS(-), EPI(-). Black solid line means EPI control group: LPS(-), EPI(+). Blue solid line represents Group A: LPS (+), EPI (-). Red solid line represents Group B: LPS(+), EPI(+), + propranolol. Green solid line represents Group C: LPS (+), EPI(+). Purple solid line represent Group D: LPS(+), EPI(+), + esmolol. We analyzed both time- and frequency-domain measures of HRV, as follows: (A) - heart rate; (B) – SDANN; (C) - SD delta; (D) - pNN50; (E) – VLF (very low frequency); (F) – LF (low frequency); (G) – HF (high frequency); and (H) - LF/HF. EPI: Epinephrine; HRV: Heart rate variability; LPS: Lipopolysaccharide; SDANN: Standard deviation of the average NN intervals; pNN50: Percentage of successive RR intervals that differ by more than 50ms.

At baseline, HR was comparable among all groups. Overall, there was a similar trend in HR change in Group D (LPS+EPI with esmolol), Group A (LPS only) and saline placebo control group. Both experimental groups (Group A and D) had significantly decreased HRs at 2 hours compared with 0.5 hour after LPS inoculation. In EPI control group, the HR remained stable at all time points. Compared with two control groups (saline placebo control and EPI control group), the HR in Group B (LPS+EPI with propranolol) was steeply decreased at the time of EPI infusion (*p* < 0.001) and further declined at 0.5 hour after LPS inoculation (*p* = 0.028), where it remained slow and stable. Moreover, at three time points after LPS inoculation, the HR was significantly decreased in Group B compared with the two control groups (saline placebo control and EPI control group) and the other three experimental groups (all p < 0.05). After EPI infusion, the HR in Group D continuously decreased until the end of experiment (*p* < 0.05; Table 1 and Figure 2A).

**TABLE 1:**
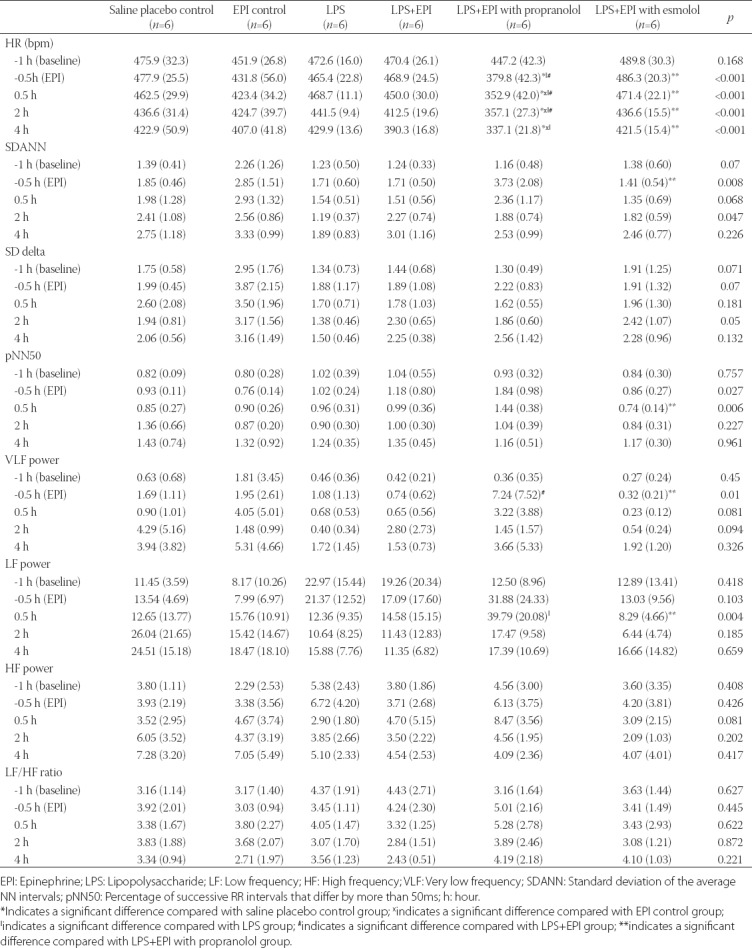
Summary of HRV parameters in LPS-induced rat sepsis model, and in relation to EPI and propranolol/esmolol administration

#### SDANN and SD delta

The changes in SDANN and SD delta between the five time points (-1, -0.5, 0.5, 2, and 4 hours) within each group were not statistically significant. At the time of EPI infusion, the SDANN in Group D (LPS+EPI with esmolol) was lower than in Group B [LPS+EPI with propranolol] (*p* = 0.047). There was no significant difference in the SD delta between groups at each time point (Table 1, Figure 2B and C).

#### pNN50

The changes in pNN50 between the five time points within each group were not statistically significant. At 0.5 hours after LPS inoculation, the pNN50 in Group D (LPS+EPI with esmolol) was significantly lower than in Group B [LPS+EPI with propranolol] (*p* = 0.015; Table 1 and Figure 2D).

#### VLF power

The changes in VLF power between the five time points within each group were not statistically significant. At the time of EPI infusion, the VLF power in Group B (LPS+EPI with propranolol) was higher than in Group C (LPS+EPI) and Group D [LPS+EPI with esmolol] (*p* = 0.048 and *p* = 0.040, respectively) (Table 1 and Figure 2E).

#### LF power, HF power, LF/HF ratio

The changes in LF power between the five time points within each group were not statistically significant. At 0.5 hour after LPS inoculation, the LF power in Group B (LPS+EPI with propranolol) was slightly higher than in Group A [LPS only] (*p* = 0.047) and significantly higher than in Group D [LPS+EPI with esmolol] (*p* = 0.015). On the other hand, there was no statistically significant difference in the HF power and LF/HF ratio between groups at each time point. Furthermore, the changes in HF power and LF/HF ratio between the five time points within each group were not statistically significant (Table 1 and Figure 2F-H).

### Cytokines

#### IL-1β

**FIGURE 3:**
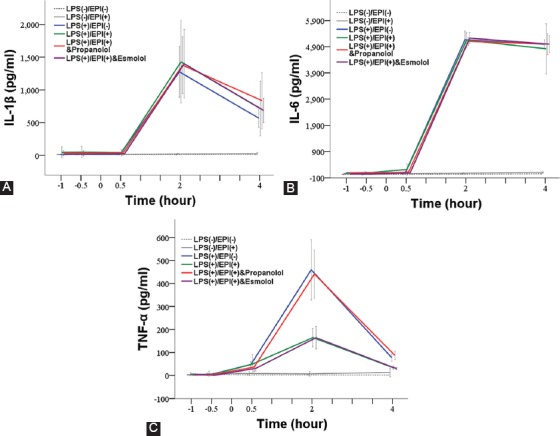
Changes in the levels of pro-inflammatory cytokines in LPS-induced rat sepsis model, and in relation to EPI and propranolol/esmolol administration. A total of 36 male Sprague-Dawley rats were randomized into two control groups and four experimental groups of six animals each. Black dotted line denotes saline placebo group: LPS(-), EPI(-). Black solid line means EPI control group: LPS(-), EPI(+). Blue solid line represents Group A: LPS (+), EPI (-). Red solid line represents Group B: LPS(+), EPI(+), + propranolol. Green solid line represents Group C: LPS (+), EPI(+). Purple solid line represents Group D: LPS(+), EPI(+), + esmolol. (A) - IL-1β; (B) - IL-6; and (C) - TNF-α. EPI: Epinephrine; HRV: Heart rate variability; LPS: Lipopolysaccharide; IL-1β: Interleukin 1 beta; IL-6: Interleukin 6; TNF-α: Tumor necrosis factor alpha.

The IL-1β levels in the control groups remained stable at all time points. In the four experimental groups, the IL-1β levels steeply increased at 2 hours after LPS inoculation (*p* ≤ 0.003 compared to 0.5 hour) and then decreased at 4 hours after LPS inoculation (*p* ≤ 0.010 compared to 2 hours). The levels of IL-1β were significantly higher in the four experimental groups compared with control groups at 2 hours (*p* < 0.001) and at 4 hours (*p* ≤ 0.048) after LPS inoculation. However, no significant difference in IL-1β levels was observed among the four experimental groups (Table 2 and Figure 3A).

**TABLE 2:**
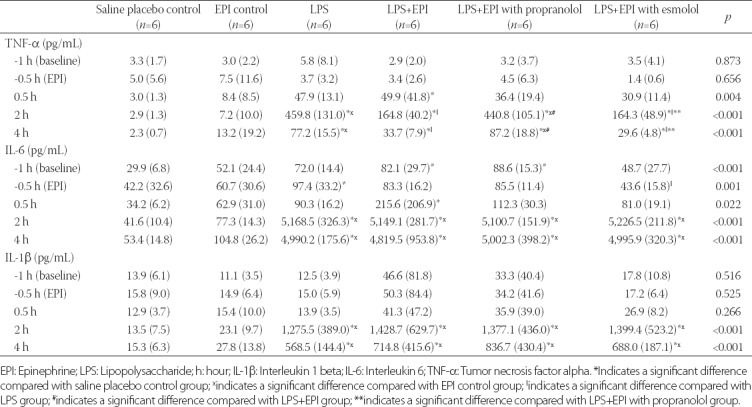
Summary of pro-inflammatory cytokine levels in LPS-induced rat sepsis model, and in relation to EPI and propranolol/esmolol administration

#### IL-6

The IL-6 levels in the control groups remained stable at all time points. The IL-6 levels in the four experimental groups sharply increased at 2 hours after LPS inoculation (*p* < 0.001 compared to 0.5 hour) and then remained stable to 4 hours after LPS inoculation. The levels of IL-6 were significantly higher in the experimental than in control groups (*p* < 0.001), but no significant difference was observed among the four experimental groups (Table 2 and Figure 3B).

#### TNF-α

The TNF-α levels in the control groups remained stable during the entire experimental period. In the four experimental groups, the TNF-α levels first increased within 2 hours of LPS inoculation, and then significantly decreased at 4 hours after LPS inoculation (*p* < 0.001 compared to 2 hours). At 2 and 4 hours after LPS inoculation, the TNF-α levels in Group A (LPS) and Group B (LPS+EPI with propranolol) were significantly higher than in control groups (*p* < 0.001); the TNF-α levels in Group C (LPS+EPI) and Group D [LPS+EPI with esmolol] were higher than in saline placebo control group (*p* = 0.047). In addition, at 2 and 4 hours after LPS inoculation, the TNF-α levels in Group A and Group B were significantly higher than in Group C and Group D (*p* < 0.001; Table 2 and Figure 3C).

### Lactate

At baseline, there was no significant difference in lactate levels among groups. At 2 hours after LPS inoculation, the lactate levels in EPI control group and Group A (LPS) were significantly increased compared with saline placebo control group (4.02 and 3.40 vs. 2.02 mmol/L, *p* < 0.001 and *p* = 0.002, respectively). The lactate levels in Group C (LPS+EPI) and Group D (LPS+EPI with esmolol) were significantly increased compared with saline placebo control group (4.56 and 4.50 vs. 2.02, both *p* < 0.001) and Group A (4.56 and 4.50 vs. 3.40, *p* = 0.011 and *p* = 0.025, respectively). At 2 hours after LPS inoculation, the lactate level in Group B (LPS+EPI with propranolol) was significantly lower than in EPI control group (2.41 vs. 4.02, *p* < 0.001), and was also lower than in Group A (2.41 vs. 3.40, *p* = 0.044), Group C (2.41 vs. 4.56, *p* < 0.001) and Group D [2.41 vs. 4.50, *p* < 0.001] (Table 3).

**TABLE 3:**
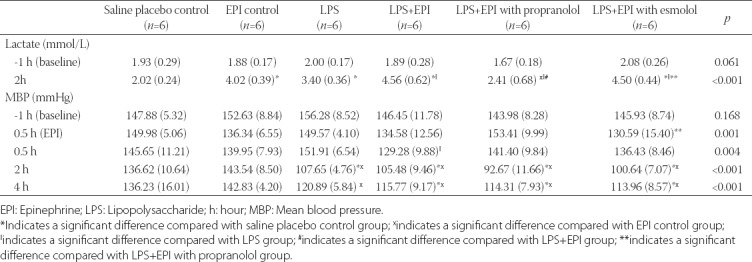
Summary of lactate and MBP values in LPS-induced rat sepsis model, and in relation to EPI and propranolol/esmolol administrationA

### MBP

**FIGURE 4:**
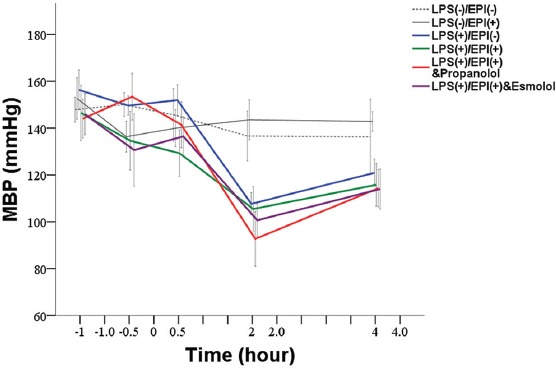
Changes in mean blood pressure in LPS-induced rat sepsis model, and in relation to EPI and propranolol/esmolol administration. A total of 36 male Sprague-Dawley rats were randomized into two control groups and four experimental groups of six animals each. Black dotted line denotes saline placebo group: LPS(-), EPI(-). Black solid line means EPI control group: LPS(-), EPI(+). Blue solid line represents Group A: LPS (+), EPI (-); Red solid line represents Group B: LPS(+), EPI(+), + propranolol. Green solid line represents Group C: LPS (+), EPI(+); Purple solid line represents Group D: LPS(+), EPI(+), + esmolol. EPI: Epinephrine; LPS: Lipopolysaccharide; MBP: Mean blood pressure.

At baseline, there was no significant difference in the MBP among groups. Immediately after LPS inoculation, the MBP was not significantly different between pair-wise groups except between Group D (LPS+EPI with esmolol) and Group B (LPS+EPI with propranolol), with a significantly lower MBP in Group D (130.59 vs. 153.41, *p* = 0.019). At 0.5 hour after LPS inoculation, a significant difference in the MBP was observed only between Group C (LPS+EPI) and Group A [LPS] (129.28 vs. 151.91, *p* = 0.010). In all four experimental groups, the MBP was significantly decreased at 2 hours after LPS inoculation compared with 0.5 hour after EPI infusion (all *p* ≤ 0.008) and was significantly lower than in saline placebo and EPI control groups (107.65, 105.48, 92.67 and 100.64 mmHg for Group A, B, C and D, respectively vs. 136.62 and 143.54 mmHg for saline placebo and EPI control, respectively; *p* < 0.001). At 4 hours after LPS inoculation, the MBP in the four experimental groups was significantly increased compared with 2 hours after LPS injection (*p* ≤ 0.010). However, the MBP in Group A was still lower than in EPI control group (120.89 vs. 142.83, *p* = 0.018). In the three groups with LPS and EPI treatments, the MBP was lower than in saline placebo and EPI control groups [MBP of 115.77, 114.31 and 113.96 mmHg in Group B, C and D, respectively vs. 136.23 and 142.83 mmHg in saline placebo and EPI control group, respectively; *p* < 0.001] (Table 3 and Figure 4).

## DISCUSSION

In this study, we investigated the effect of EPI on cardiovascular and immune response in LPS-induced rat sepsis model. Our results indicated that LPS inoculation of rats led to impaired function of the autonomic nervous system, as indicated by decreased HRV and increased levels of pro-inflammatory cytokines TNF-α, IL-1β, and IL-6. Compared to saline placebo control group, the HRs were decreased in all experimental groups, especially in Group B (LPS+EPI with propranolol). However, LF and HF were decreased in Group D (LPS+EPI with esmolol) but not in Group B (LPS+EPI with propranolol). There were significant differences in the HF between Group A (LPS) and saline placebo control group after 2 hours of LPS inoculation. The decrease in HF in Group A suggested altered parasympathetic (vagal) activity. In contrast, such reduction in HF was not observed in Group C (LPS+EPI), indicating a positive effect of EPI on HRV. Moreover, the antecedent EPI infusion in Group C resulted in a significantly lower increase of TNF-α compared with Group A. In Group B, it seems that propranolol (a nonselective β1- and β2-blocker) blocked the effect of EPI, since the LF and HF were significantly increased compared with Group A. On the other hand, the LF and HF in Group D were decreased as in Group C, indicating that esmolol (a selective β_1_-blocker) did not block the effect of EPI. At 2 and 4 hours after LPS inoculation, the TNF-α levels in Group A and B were significantly higher than in Group C and D. Thus, EPI may inhibit TNF-α production by acting on β2-ARs.

In healthy volunteers, intravenous administration of *E. coli* endotoxin was associated with loss of the variability by all measures of HRV (time domain, frequency domain, and measure of regularity) [22]. Similarly in another study on healthy subjects, LPS administration affected all measured HRV parameters (SDANN, pNN50, and RMSSD; HF, LF, LF/HF, and VLF), while antecedent EPI infusion was associated with reductions in HRV parameters reflecting parasympathetic activity (i.e. pNN50, RMSSD, and HF) [12].

Internal homeostasis after injury or infection is balanced by activation of anti-inflammatory and pro-inflammatory pathways. Endotoxin-induced systemic inflammation is attenuated by acetylcholine, a principle vagal neurotransmitter [23]. Acetylcholine significantly inhibited the release of cytokines, including IL-1β, IL-6, IL-18, and TNF in LPS-stimulated macrophage culture. Goldstein et al. [24] showed that while sympathetic modulation of cardiac activity decreased during endotoxin-mediated shock in rabbits, the sympathomedullary response did not change. Sympathovagal imbalance has been associated with a higher risk of death in critically ill patients, increased incidence of sepsis in patients with systemic inflammatory response syndrome, pain hypersensitivity (hyperalgesia), and pathophysiology of chronic heart failure (CHF), among other conditions [25-27]. Furthermore, the trigeminocardiac reflex (TCR), a well-established brainstem reflex, strongly correlated with the depth of anesthesia. Pretreatment with anticholinergic drugs to block the action of acetylcholine can reduce the potential of the vagus nerve, thereby reducing the prevalence of TCR [28]. Although they recommend to administer anticholinergic drugs only in the case of persistent asystole or repetitive TCR, patients with sepsis and TRCs should carefully consider possible treatment options.

*In vitro* studies using human whole blood showed that both norepinephrine (NE) [13,29] and EPI [14,30,31] inhibit endotoxin-induced immune cell production of pro-inflammatory cytokines. Moreover, short term pre-exposure of healthy subjects to EPI before injection of low-dose endotoxin decreased the secretion of the pro-inflammatory cytokine TNF-α and stimulated the production of the anti-inflammatory IL-10 [31]. In our previous study [2], the concentrations of IL-6 and TNF-α were the highest 2 hours after *E. coli* inoculation in immunocompromised rats and then subsequently decreased at 6 hours, while IL-1β levels stayed approximately the same. In the current study, the concentrations of TNF-α, IL-1β, and IL-6 were all higher after LPS intravenous infusion in rats, peaking at 2 hours.

The effects of catecholamines on cytokine production appear to be mediated by β-ARs. For example, the inhibition of LPS-stimulated TNF-α production by NE in whole blood from healthy subjects and patients with CHF was abolished by the β-blocker bisoprolol [13]. Another study investigating the role of β1- and β2-ARs in the production of total monocyte TNF receptor (moTNFR) and TNF in LPS-treated whole blood from healthy subjects showed that EPI action was mediated by β2-ARs [32]. Moreover, β2-ARs in regulatory lymphocytes were suggested to be critical for the anti-inflammatory activity of the vagus nerve, as demonstrated in β2-knockout mice [1]. In the present study, the nonselective β-AR blocker propranolol blocked the inhibitory effect of EPI on TNF-α production, whereas the β1-AR blocker esmolol did not. Although our results suggest that EPI inhibited the production of TNF-α through β2-ARs, these interactions may be more complex, since propranolol also blocks β3-ARs [33]. All three β-ARs (β1, β2, and β3) are expressed on cardiac myocytes [8], however, the role of β3-AR in LPS-induced immune response is not completely clear. For example, by evaluating mice lacking β1- and β2-ARs, Walker-Brown and Roberts [8] showed that β3-AR does not have a significant role in in regulating LPS-mediated mortality and inflammation [8]. In addition to TNF-α, norepinephrine and EPI were found to inhibit LPS-induced IL-6 [29] and IL-1β [31] production in adult humans. In our study, LPS was able to promote IL-6 and IL-1β production in rat sepsis model (Figure 3). However, EPI treatment did not inhibit IL-6 and IL-1β production. It is not clear whether this discrepancy is due to the differences between rats and humans, and it warrants further investigation.

Sepsis is known to be closely related to systemic inflammation, multiple organ dysfunction, and mortality. The results of a large-scale and state-wide population trauma study further found that increased severity of injury is an important independent predictor of traumatic sepsis and is associated with mortality [34]. Patients with sepsis have higher mortality and ICU admission rates compared with patients without sepsis (23.1% vs. 7.6% and 94% vs. 40%, respectively). The progression of sepsis to multiple organ dysfunction is thought to be caused by an uncontrolled release of inflammatory factors and the study by Gino et al. suggested that the carotid body plays a protective role during sepsis [3]. LPS administration significantly increased the instantaneous heart frequency and minute ventilation volume. However, LPS-induced tachypnea was suppressed in rats with bilateral carotid neurotomy. At the same time, LPS-induced TNF-α and EPI upregulation was effectively boosted in the rats with bilateral carotid neurotomy, suggesting that the carotid body may play an important role in regulating EPI inhibition of LPS-induced TNF-α expression [3].

Regarding the effect of EPI on hemodynamics, we showed that the MBP significantly decreased in experimental groups 2 hours following LPS infusion. Therefore, in the hyperdynamic phase of sepsis, EPI seems to be ineffective in increasing and maintaining blood pressure and it even led to increased lactate levels.

Since EPI is used in the treatment of septic shock, a number of studies investigated *in vivo* effects of catecholamines using animal models. In domestic pigs, during cardiopulmonary resuscitation, EPI reduced cerebral perfusion through its agonist action at α1-AR [35]. In a long-term rodent model of sepsis, the pressor response to intravenous NE was significantly decreased in septic animals, while response to vasopressin was preserved [36]. In another rat model of LPS-induced septic shock, simultaneous early fluid resuscitation and NE treatment had a superior effect against septic shock-induced lung injury over traditional fluid strategy [37].

It should be emphasized here that the applicability of our findings toW clinical practice may be affected by the presence of certain pathological conditions. For instance, hypertension, diabetes mellitus, and renal failure are well-known cardiovascular risk factors and therefore can play a role in pathophysiological processes related to the cardiovascular system [38].

There are several limitations to our study. First, we investigated the effects of EPI on sepsis-induced changes only in a rat model under general anesthesia, and not in patients. Moreover, the number of animals included was small, and a larger sample size should be used to increase the statistical power of study. Second, although LPS is able to induce sepsis, LPS is only one of the substances secreted by bacteria. Thus, this LPS effect is not completely equal to the effects of bacteria in clinical practice. Third, we did not test whether a specific β2-AR blocker inhibits the effect of EPI on cytokine production, which would provide additional evidence for the role of β2-ARs in mediating EPI effects during sepsis. Finally, for the accurate assessment of sympathovagal balance, in addition to HRV, endogenous NE and acetylcholine should be evaluated. In this study, we did not perform such analysis due to the lack of appropriate equipment.

## CONCLUSION

In conclusion, LPS inoculation in rats caused significant changes in HRV and promoted inflammation, indicating imbalance of the autonomic nervous system. The antecedent EPI infusion increased HRV and inhibited the production of TNF-α possibly via the β_2_-AR. Because EPI can induce both pro- and anti-inflammatory effects, the balance between these opposing effects might be achieved by controlling the quantity and timing of EPI administration.
